# Inhibition of the glucocorticoid receptor attenuates proteinuric kidney diseases in multiple species

**DOI:** 10.1093/ndt/gfad254

**Published:** 2023-11-30

**Authors:** Eleni Stamellou, Shipra Agrawal, Florian Siegerist, Marc Buse, Christoph Kuppe, Tim Lange, Eva Miriam Buhl, Jessica Alam, Thiago Strieder, Peter Boor, Tammo Ostendorf, Hermann-Josef Gröne, Jürgen Floege, William E Smoyer, Nicole Endlich, Marcus J Moeller

**Affiliations:** Division of Nephrology and Clinical Immunology, RWTH Aachen University, Aachen, Germany; Institute of Pathology and Electron Microscopy Facility, RWTH University of Aachen, Aachen, Germany; Department of Nephrology, Medical School, University of Ioannina, Ioannina, Greece; Division of Nephrology and Hypertension, Department of Medicine, Renaissance School of Medicine, Stony Brook University, Stony Brook, NY, USA; Department of Anatomy and Cell Biology, University Medicine Greifswald, Greifswald, Germany; Division of Nephrology and Clinical Immunology, RWTH Aachen University, Aachen, Germany; Division of Nephrology and Clinical Immunology, RWTH Aachen University, Aachen, Germany; Institute of Experimental Medicine and Systems Biology, RWTH Aachen University, Aachen, Germany; Department of Anatomy and Cell Biology, University Medicine Greifswald, Greifswald, Germany; Institute of Pathology and Electron Microscopy Facility, RWTH University of Aachen, Aachen, Germany; Division of Nephrology and Clinical Immunology, RWTH Aachen University, Aachen, Germany; Division of Nephrology and Clinical Immunology, RWTH Aachen University, Aachen, Germany; Division of Nephrology and Clinical Immunology, RWTH Aachen University, Aachen, Germany; Institute of Pathology and Electron Microscopy Facility, RWTH University of Aachen, Aachen, Germany; Division of Nephrology and Clinical Immunology, RWTH Aachen University, Aachen, Germany; Institute of Pharmacology, Philipps-University of Marburg, Marburg, Germany; Division of Nephrology and Clinical Immunology, RWTH Aachen University, Aachen, Germany; Center for Clinical and Translational Research, Abigail Wexner Research Institute at Nationwide Children's Hospital, and Department of Pediatrics, The Ohio State University, College of Medicine, Columbus, OH, USA; Department of Anatomy and Cell Biology, University Medicine Greifswald, Greifswald, Germany; NIPOKA, Greifswald, Germany; Division of Nephrology and Clinical Immunology, RWTH Aachen University, Aachen, Germany

**Keywords:** FSGS, glucocorticoid receptor (GR), MCD, mifepristone, proteinuria

## Abstract

**Background:**

Glucocorticoids are the treatment of choice for proteinuric patients with minimal change disease (MCD) and primary focal segmental glomerulosclerosis (FSGS). Immunosuppressive as well as direct effects on podocytes are believed to mediate their actions. In this study, we analyzed the anti-proteinuric effects of inhibition of the glucocorticoid receptor (GR) in glomerular epithelial cells, including podocytes.

**Methods:**

We employed genetic and pharmacological approaches to inhibit the GR. Genetically, we used Pax8-Cre/GR^fl/fl^ mice to specifically inactivate the GR in kidney epithelial cells. Pharmacologically, we utilized a glucocorticoid antagonist called mifepristone.

**Results:**

Genetic inactivation of GR, specifically in kidney epithelial cells, using Pax8-Cre/GR^fl/fl^ mice, ameliorated proteinuria following protein overload. We further tested the effects of pharmacological GR inhibition in three models and species: the puromycin aminonucleoside–induced nephrosis model in rats, the protein overload model in mice and the inducible transgenic NTR/MTZ zebrafish larvae with specific and reversible podocyte injury. In all three models, both pharmacological GR activation and inhibition consistently and significantly ameliorated proteinuria. Additionally, we translated our findings to humans, where three nephrotic adult patients with MCD or primary FSGS with contraindications or insufficient responses to corticosteroids were treated with mifepristone. This treatment resulted in a clinically relevant reduction of proteinuria.

**Conclusions:**

Thus, across multiple species and proteinuria models, both genetic and pharmacological GR inhibition was at least as effective as pronounced GR activation. While the mechanism remains perplexing, GR inhibition may be a novel and targeted therapeutic approach to treat glomerular proteinuria potentially bypassing adverse actions of steroids.

Key Learning Points **What was known:**

- Glucocorticoids are the preferred treatment for proteinuric patients with minimal change disease and focal segmental glomerulosclerosis.
- Their immunosuppressive and direct effects on podocytes are believed to mediate their therapeutic actions.

**This study adds:**

- Both genetic and pharmacological inhibition of the glucocorticoid receptor (GR) effectively reduced proteinuria in various animal models and human patients.
- GR inhibition presents a potential alternative therapeutic approach for treating glomerular proteinuria.

**Potential impact:**

- GR inhibition may serve as a novel therapeutic approach to reduce proteinuria in diseases characterized by podocyte injury, which might enable avoidance of steroid side effects.

## INTRODUCTION

Glucocorticoids have been the therapeutic mainstay in many glomerular diseases for more than 70 years [1, 2]. However, even though vast clinical experience has been gained, the exact mechanisms by which glucocorticoids elicit their renoprotective effects are poorly understood. In general, the therapeutic benefits from glucocorticoids have been attributed to the prominent immunosuppressive effects; this concept supported use of other immunosuppressive drugs in glomerular diseases. As glucocorticoids are particularly effective in primary podocytopathies such as minimal change disease (MCD) or focal segmental glomerulosclerosis (FSGS), additional and direct effects of glucocorticoids on podocytes have been reported, although definitive *in vivo* proof for such effects is still lacking [3–7].

Clinically, glucocorticoids have been found to be essential immunosuppressive regimens. This is particularly true in rapidly progressive glomerulonephritis (RPGN), e.g. ANCA vasculitis, lupus nephritis or anti-glomerular basement membrane (GBM) disease, all of which are characterized by segmental glomerular necrosis and parietal epithelial cell (PEC) activation and proliferation [8–10]. Glucocorticoid receptors (GR) are expressed in renal epithelial cells including podocytes and PECs. We have previously shown in the murine RPGN (anti-GBM) model that glucocorticoids mediate their beneficial effects via direct actions on PECs rather than by systemic immunosuppressive effects, resulting in reduced PEC activation, fewer cellular crescents and lower proteinuria [11]. Surprisingly, we have noted that even glucocorticoid antagonism, which did not affect the immune system, had similar beneficial effects in PECs and on the outcome of anti-GBM disease [11].

Up to now, glucocorticoids remain the treatment of choice in proteinuric glomerular diseases; however, given their serious adverse effects, there is a need for new therapies beyond GR activation. We have previously identified glucocorticoid antagonism as a new therapeutic approach in crescentic nephritis [11]. In the present work, we wanted to study GR inhibition as a new therapeutical approach in proteinuric glomerular disease. We have used three different models in different species. First, we used the puromycin aminonucleoside nephrosis (PAN) model, i.e. the most commonly employed rat model of human MCD [6, 12]. Next we validated our results in the protein overload–induced nephropathy model in mice induced by daily injections of large amounts of bovine serum albumin (BSA). This model was conceived as a model for MCD because it induces reversible nephrotic-range proteinuria [13]. Third, we also assessed the transgenic NTR/MTZ model of podocyte injury in larval zebrafish [14]. Finally, and of major clinical relevance, we report the first clinical data in three nephrotic patients with primary FSGS or MCD, all treated successfully on a compassionate-use basis with the partial GR antagonist, mifepristone [15].

## MATERIALS AND METHODS

### Humans

Ethical review and approval was not required for the study on human participants, in accordance with the local legislation and institutional requirements. Informed consent for an off-label, compassionate-use therapy was obtained from all individual patients.

### Animal studies

#### Mice and rats

All procedures involving animals were approved by the institutional and local legal authorities (Az 81–02.04.2020.A299). Animals were held in rooms with constant temperature and humidity and 12-h/12-h light cycles, and had *ad libitum* access to drinking water (ozone treated and acidified) and standard chow. Pax8-Cre mice were crossed with GR^fl/fl^ mice and bred on an SV129 background for at least eight generations. Matched littermates were used as controls for all experiments. The protein-overload model was induced by daily intraperitoneal injections of BSA, 15 mg/kg body weight (Sigma Aldrich A9430). Serum and 8-h urine collections using metabolic cages were obtained during the course of the model. FVB/N wild type mice were treated with intraperitoneal injections of prednisolone (2.5 mg/kg per day; Merck), mifepristone (20 mg/kg per day; Sigma-Aldrich, St Louis, MO, USA) or vehicle (NaCl 0.9%). We used both male and female mice at an equal ratio. Studies in the PAN-nephrosis model were approved by the Institutional Animal Care and Use Committee at Nationwide Children's Hospital (NCH), Columbus, OH, USA, and performed at NCH. A single 50 mg/kg dose of PAN was administrated intravenously on Day 0 to male Wistar rats weighting ∼150–200 g to induce nephrosis. The control group received saline. Groups of PAN-injected rats then received vehicle solution intraperitoneally (i.p.) (sham controls), or nine daily doses of methylprednisolone (15 mg/kg i.p., Solu-Medrol, Pfizer Inc., New York, NY, USA) or daily mifepristone (2.5 mg/kg i.p., Sigma-Aldrich, St Louis, MO, USA) starting on Day 0. Urine collections were performed at baseline (prior to injury) and daily thereafter until the rats were sacrificed on Day 11, i.e. the time of expected peak proteinuria.

### Zebrafish model

Zebrafish (*Danio rerio*) were maintained as described previously [16]. The *Tg(nphs2: GAL4); Tg(UAS: Eco.nfsB-mCherry*) strain expresses the NTR-mCherry fusion protein under podocyte-specific control of the *nphs2* promotor [14]. Larval zebrafish were kept at 28.5°C in E3 solution. Zebrafish larvae were pretreated for 5 h with either 100 nM mifepristone (Sigma-Aldrich, St Louis, MO, USA) or 100 nM dexamethasone (Merck, Darmstadt, Germany). After that, the medium was exchanged to 100 µM metronidazole (MTZ) (Sigma-Aldrich, St Louis, MO, USA) in 0.1% DMSO-E3 medium or medium alone both spiked with either 100 nM dexamethasone (Merck, Darmstadt, Germany) or 100 nM mifepristone (Sigma-Aldrich, St Louis, MO, USA) at 28.5°C in the dark. The development of pericardial and periocular edema was evaluated under a stereomicroscope (Stemi SV11, 2.5X/0.075 objective Neofluar, Carl Zeiss Microimaging, Jena, Germany) at 48 h after the start of treatment.

### Zebrafish immunofluorescence

Zebrafish larvae (6 days past fertilization) were fixed in 2% paraformaldehyde in 1× phosphate-buffered saline (PBS) for 3 h at room temperature. The larvae were then incubated in 30% sucrose in 1× PBS at 4°C overnight, embedded in Tissue Tek (Sakura, Staufen, Germany) and snap-frozen in liquid nitrogen. Transversal cross sections (10 µm) were cut with a CM1950 cryotome (Leica Biosystems, Nussloch, Germany). After blocking [2% fetal bovine serum (FBS), 2% BSA, 0.2% fish gelatin in 1× PBS] for 1 h, the sections were incubated with a polyclonal rabbit anti-zebrafish nephrin primary antibody (1/2000, Innovagen, Lund, Sweden) at 4°C overnight. After three washes with 1× PBS, the sections were incubated with an Alexa 647 conjugated goat anti-rabbit F(ab) antibody fragment (1/300, Jackson Immuno Research, West Grove, PA, USA) for 1 h at 4°C. The slides were then washed three times in 1× PBS, incubated with 0.013 mg/mL Hoechst (Sigma Aldrich, St Louis, MO, USA) and mounted in mowiol for microscopy (Carl Roth, Karlsruhe, Germany). The stained sections were imaged using a TCS SP5 confocal laser scanning microscope (Leica Microsystems, Wetzlar, Germany) with a 63× (1.3 NA, oil immersion) objective.

### Assessment of renal function and abuminuria

Serum and urine creatinine were determined using the test kit Creatinine Plus Version 2 (Roche Diagnostics, Basel, Switzerland), and blood urea nitrogen was analyzed using a Hitachi 9-17-E Autoanalyzer (Hitachi, Frankfurt am Main, Germany). Albumin concentrations in serum und urine were measured using a competitive two-step enzyme immunoassay (MP BiomedicalsEschwege, Germany). In the PAN nephrosis model: proteinuria [urine protein:creatinine ratios (UPCR)] were measured by Antech Diagnostics GLP (Morrisville, NC, USA), a fully compliant Good Laboratory Practice–regulated service, as reported previously [17].

### Electron microscopy

Small pieces of kidney cortex were fixed in Karnovsky solution and embedded in Epon (Serva, Heidelberg, Germany). Samples were washed in 0.1 M Soerensen's phosphate buffer (Merck, Darmstadt, Germany), post-fixed in 1% OsO4 (Roth, Karlsruhe, Germany) in 0.25 M sucrose buffer (Merck, Darmstadt, Germany) and dehydrated by ascending ethanol series (30%, 50%, 70%, 90% and 100%) for 10 min each. The last step was repeated three times. Dehydrated specimens were incubated in propylene oxide (Serva, Heidelberg, Germany) for 30 min, in a mixture of Epon resin (Serva, Heidelberg, Germany) and propylene oxide (1:1) for 1 h and finally in pure Epon for 1 h. Epon polymerization was performed at 90°C for 2 h. Contrast of ultrathin sections was enhanced by staining with 0.5% uranyl acetate and 1% lead citrate (both EMS, Munich, Germany). Ultrathin sections were examined with a transmission electron microscope ZEISS Leo 906 (Carl Zeiss, Oberkochen, Germany) at 60 kV by a magnification from 3597–6000×.

### Light microscopy

For light microscopy, 4% buffered formalin-fixed kidney fragments were dehydrated, embedded in paraffin and stained with periodic acid–Schiff (PAS).

### Immunoperoxidase histochemistry

Immunohistochemistry was performed on 2 μm paraffin sections. Sections were blocked with an avidin/biotin blocking kit (Vector Laboratories, Burlingame, CA, USA) and 3% H_2_O_2_. The sections were subjected to microwave antigen retrieval in Antigen Unmasking Solution (Vector Laboratories, CA, USA) followed by incubation with the primary and secondary antibodies. The primary antibody was anti-desmin (Rabbit mAB#5332, Cell Signaling). As secondary antibody, we used biotinylated goat anti–rabbit (Vector Laboratories, CA, USA). Detection was carried out with the Vectastain ABC Kit (Vector Laboratories) with the use of peroxidase as label, 3,3′-diaminobenzidine as substrate and nickel chloride enhancement.

Desmin staining in podocytes was assessed via a semiquantitative scoring; Score 0: absent staining or staining <5% of the assessed area; Score I: 5%–25% stained area; Score II: 25%–50% stained area; Score III: 50%–75% stained area; Score IV: >75% stained area. Glomerular desmin expression was quantified in 25 glomeruli and expressed as a ratio of stained glomeruli/total area of glomerulus.

### Podocyte exact morphology measurement procedure

The staining protocol was performed as already described. Briefly, after deparaffinization in xylene and rehydration in a descending ethanol series, all sections were boiled in a pressure cooker in Tris EDTA buffer (10 mmol/L Tris, 1 mmol/L EDTA, pH 9, 0.1% Tween 20) for antigen retrieval. The slides were incubated for 1 h in blocking solution (1% FBS, 1% BSA, 0.1% fish gelatin, 1% normal goat serum in PBS). The primary antibodies (1:75 in blocking solution, rabbit anti‐podocin, Sigma-Aldrich, and mouse anti‐synaptopodin 1:100 in blocking solution, Progen, Heidelberg, Germany) were incubated at 4°C on the slides overnight. On the next day, the slides were washed three times in 1× PBS, followed by a blocking step for 45 min and incubation with the secondary antibodies (all 1:500 in blocking solution: Cy3‐conjugated anti-mouse, Jackson Immuno Research, Hamburg, Germany and Alexa Fluor 488‐conjugated anti‐mouse IgG, Jackson Immuno Research) at 4°C for 1 h. After three washes in PBS, the slides were incubated in purified water and mounted in Mowiol (Carl Roth) using high-precision cover glasses (Paul Marienfeld GmbH). Three-dimensional structured illumination microscopy (3D-SIM): for 3D-SIM, 19 images (z-stack) were taken from 3-µm thick tissue sections by a N-SIM Nikon microscope with a 100× silicone objective. The podocyte exact morphology measurement procedure was performed as published by measuring the filtration slit density, i.e. the length of the filtration slit per area [18]. This was performed in an automated manner on the maximum intensity projection of the 3D-SIM image. For each group of animals, 20 glomeruli were measured.

### qRT-PCR

#### Mice

RNA isolation, RNA purity determination, cDNA synthesis and RT-PCR were performed as described previously [19]. The primer sequences are listed in Table 1. Glyceraldehyde-3-phosphate dehydrogenase cDNA amplification was used as an internal standard.

**Table 1:** Primer sequences for SYBR green-based real-time RT-PCR.

Gene	Forward	Reverse
*gapdh*	GGCAAATTCAACGGCACAGT	AGATGGTGATGGGCTTCCC
*ccl5*	AGTGCTCCAATCTTGCAGTCG	CACTTCTTCTCTGGGTTGGCA
*cxcl19*	TGATCTTCTTTTCCCATTCTTTCA	CGGAGATCAAACCTGCCTAGA
*cxcr4*	ACCTCTACAGCAGCGTTCTCATC	TGTTGGTGGCGTGGACAATA
*ccr6*	ACAGAGCCATCCGAGTCGTGAT	CTGGTGTAGGCGAGGACTTTCT
*ccl20*	GTGGGTTTCACAAGACAGATGGC	CCAGTTCTGCTTTGGATCAGCG
*nphs2*	GGCCCTGGGCTGATGTTTTA	GAGCAATGCGTTTCCTGTCC
*foxo3b*	TCACTGGAACAAAGCAGTCCA	GGCATATCATCCAGTGCTTGC
*eef1a1l1*	AAGGAGGGTAATGCTAGCGG	GGGCGAAGGTCACAACCATA
*zgc:158 463*	TTACCCCAGGCTCGGAAAAC	CGGGAAGGTCTTTGAACCCA

#### Zebrafish

RNA isolation with TRI Reagent (Sigma-Aldrich, St Louis, MO, USA) was performed according to the manufacturer’s protocol in 20 randomly picked zebrafish larvae subsequent to assessment of edema. Reverse transcriptase reaction was performed with QuantiTect Reverse Transcription Kit (Qiagen, Hilden, Germany) followed by qRT-PCR with *nphs2* as target-gene and *eef1a1l1* and *zgc:158 463* (Table 1) as reference genes with iQ SYBR Green Supermix mastermix (Bio-Rad, Hercules, CA, USA) on a Bio-Rad iCycler Thermal Cycler with iQ5 Multicolor Real-Time PCR Detection System (Bio-Rad, Hercules, CA, USA). Each qRT-PCR run was performed in triplicate. Data analysis was performed using the ΔΔCt method and stated as normalized fold expression compared with the control group (0.1% DMSO).

### Statistics

Statistical analyses were performed with GraphPad Prism v8 software. All values are expressed as means ± standard deviation (SD). For a comparison of two groups, either a *t*-test, or a two-tailed Mann–Whitney *U* test in the case of non-normally distributed values, were used. Comparison of several groups was performed using analysis of variance (ANOVA); *post hoc* Tukey correction was used for multiple comparisons. Values of *P* < .05 were considered significant. All analyses were performed in a blinded fashion.

## RESULTS

### Genetic manipulation/inactivation of glucocorticoid signaling

#### The GR is expressed in podocytes in patients with FSGS

**Figure 1:**
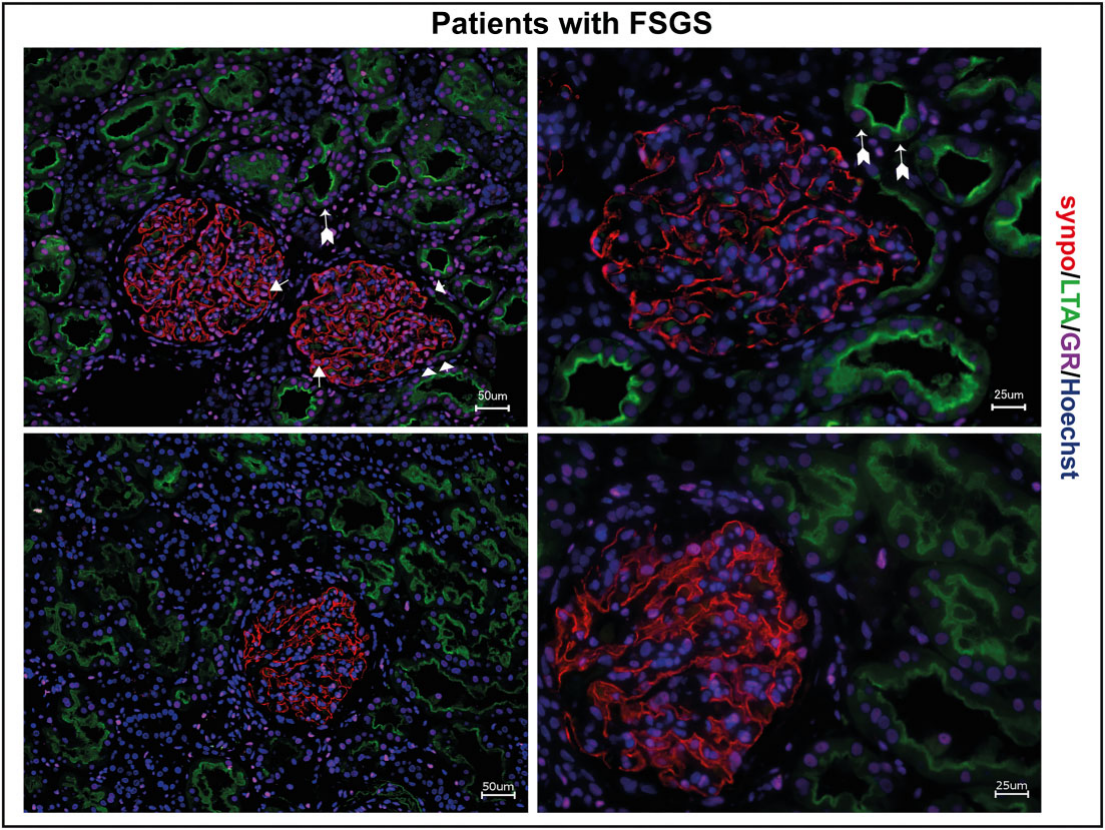
GR expression in human MCD/FSGS. Immunofluorescence staining of human kidney biopsies of two patients with FSGS. GR expression (magenta) is visible in all glomerular cells, including podocytes (synaptopodin; red, arrow) and parietal epithelial cells (arrowheads), but also in proximal tubule cells (LTA; green, arrows with tails). Scale bars 25 μm and 50 μm.

It has been shown previously that under physiological conditions, the GR is expressed ubiquitously in the nuclei of all human glomerular cells, including PECs and podocytes. Furthermore, GR expression was detectable in kidney biopsies obtained from patients with FSGS. Notably, GR was specifically localized in PECs, podocytes, and proximal tubule cells (Fig. 1A).

#### Protein overload in Pax8-Cre/GR^fl^^/fl^ mice

**Figure 2:**
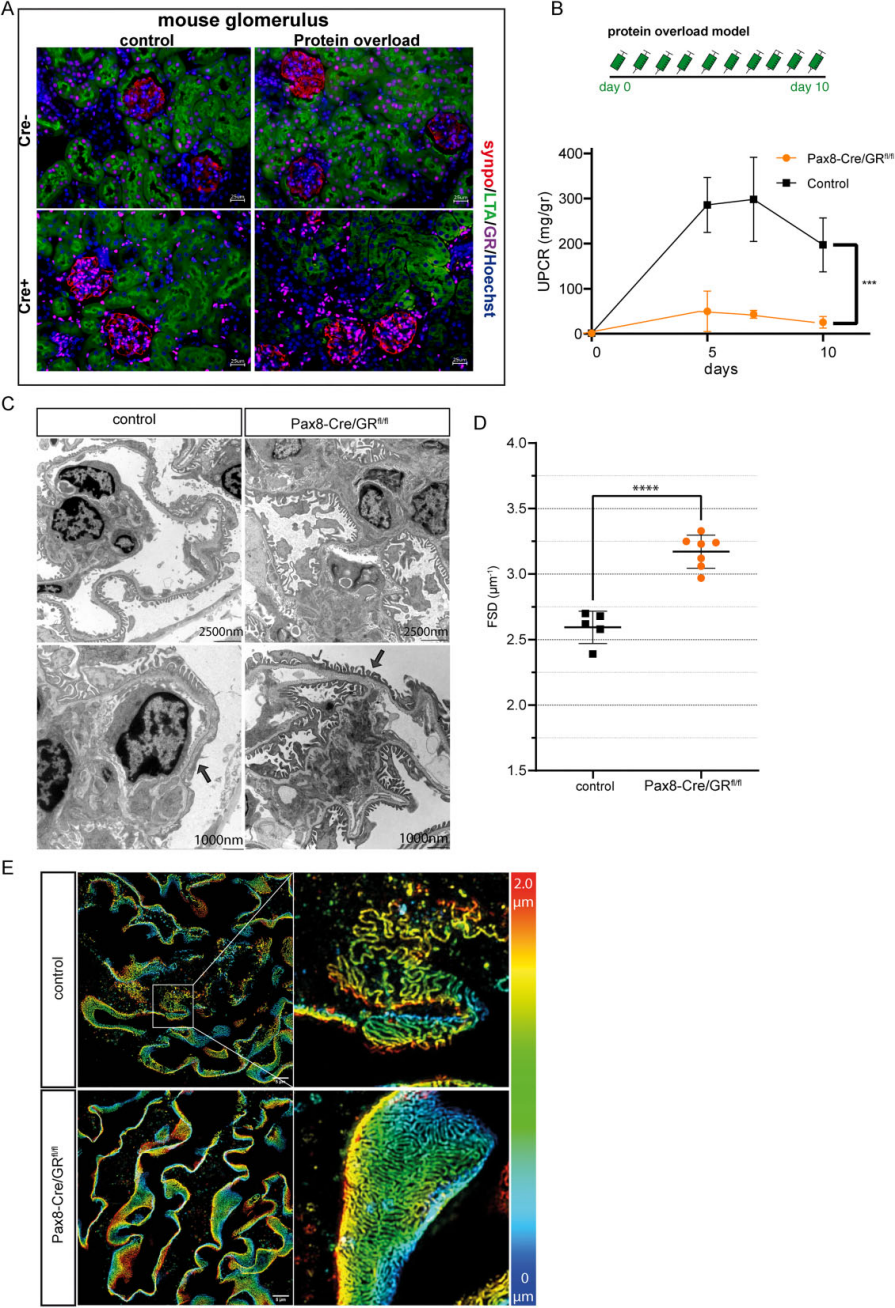
Genetic Inactivation of glucocorticoid signaling and protein overload in these mice. (**A**) Immunofluorescence costaining of GR (magenta), podocyte marker synaptopodin (synpo; red), proximal tubule cells (LTA; red) and DNA (Hoechst; blue) confirmed the selective deletion of GR in renal epithelial cells. (**B**) Schme of the protein overload model. Proteinuria was induced by bovine albumin injections (15 mg/g/body weight daily for 10 days (*n* = 6 per group) (upper). UPCR in mg/g creatinine in wildtype mice (control) and transgenic mice (ko) following protein overload (*n* = 6 per group) (lower graph). (**C**) Transmission electron microscopy from wildtype control mice and transgenic mice with protein overload (Day 10). There is reduced podocyte effacement in transgenic mice compared with wildtype controls. (**D**) Quantification of slit diaphragm density width in wildtype control and transgenic mice. Data are expressed as means ± SD. ****P < .0001 by Student's *t*-test. (**E**) Images of 3D-reconstructed SIM volumes showing the spatial aspect of the slit diaphragm on the capillary loops in control and ko mice. The same colors indicate the same Z-position within the total Z-volume of 4.5 µm. Data are expressed as means ± SD. ***P < .001, ****P < .0001 by *t*-test and two-way ANOVA.

The GR was specifically inactivated in kidney epithelial cells in Pax8-Cre/GR^fl^^/fl^ (Fig. 2A). Pax8-Cre^t/wt^/GR^fl/fl^ mice and Pax8-Cre^wt/wt^/GR^fl/fl^ controls were subjected to the protein-overload model (Fig. 2B). Genetic inactivation of the GR attenuated proteinuria (Fig. 2B) significantly already on Day 2. Podocyte injury was also attenuated in GR knock-out mice in comparison with controls (Fig. 2C). Quantification of podocyte effacement was performed via slit diaphragm density, measured using ultra-high-resolution microscopy (Podocyte Exact Morphology Measurement Procedure) [18, 20]. GR-transgenic mice showed a significantly lower average filtration slit density compared with control mice (Fig. 2D and E). No difference was observed between male and female mice (Supplementary data, Fig. S1A). Renal functional parameters, serum albumin and cholesterol were not different between the two groups (Supplementary data, Fig. S1B). Light microscopy did not reveal any difference between transgenic and control mice (Supplementary data, Fig. S1C).

### Pharmacological inhibition

#### PAN model

**Figure 3:**
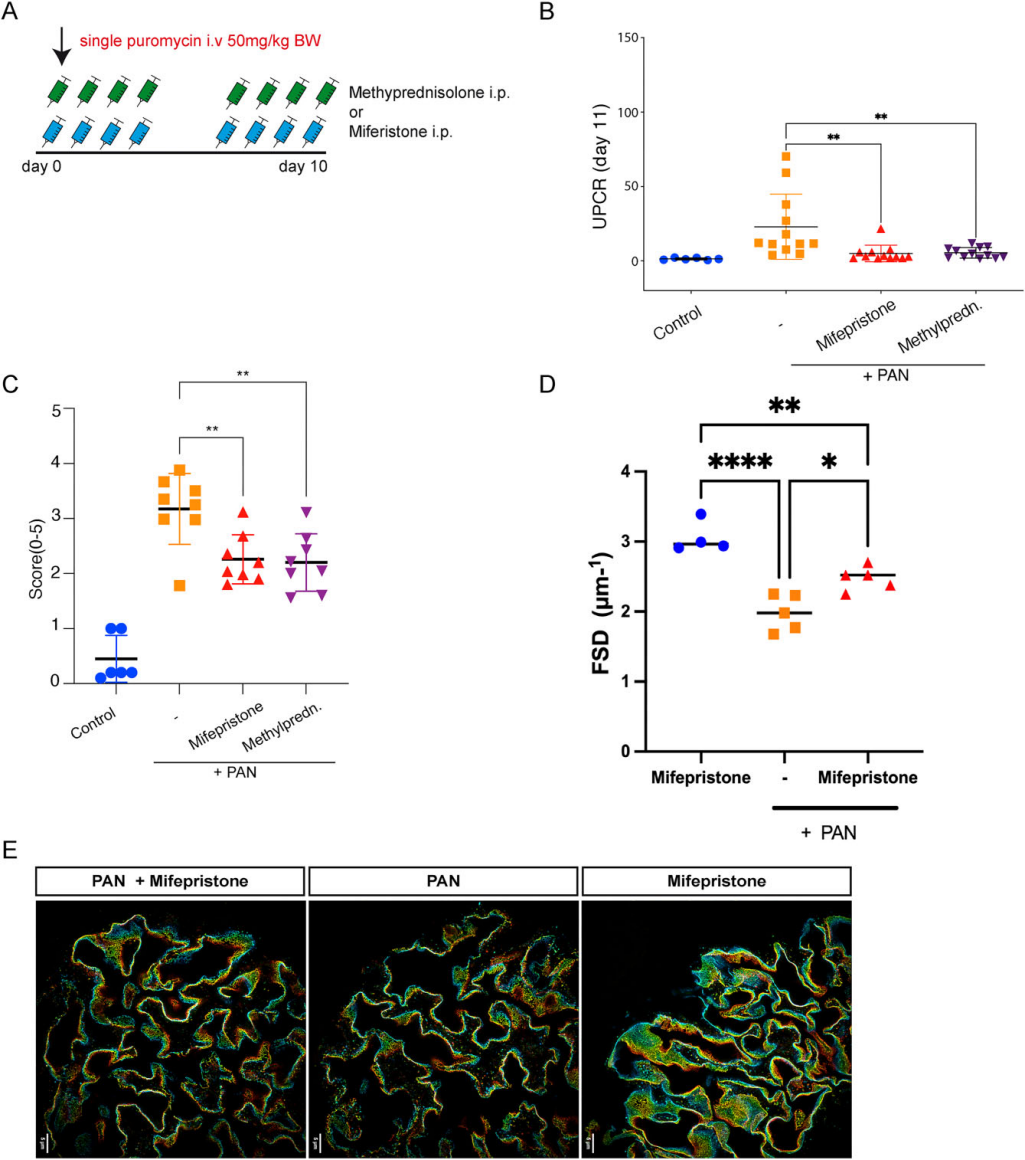
Pharmacological inhibition in PAN model. (**A**) Scheme of the PAN model. Proteinuria in rats was induced by a single intravenous (i.v.) puromycin aminonucleoside injection at 50 mg/kg body weight. The rats were then treated for 9 days with either methylprednisolone or mifepristone by i.p. injection (*n* = 6–12) (**B**) UPCR in mg/g creatinine on Day 11 in PAN, healthy control rats and treated rats. (**C**) PAS staining in methylprednisolone-treated, mifepristone-treated and untreated PAN rats showing no evidence of FSGS. (**D**) Histological quantification of glomerular desmin expression. (**E**) Quantification of slit diaphragm density width in PAN rats treated or not with mifepristone. Mifepristone treatment resulted in increased slit diaphragm density compared with vehicle treated rats. Data are expressed as means ± SD. ****P < .0001 by Student's *t*-test. (**G**) Images of 3D-reconstructed SIM volumes showing the spatial aspect of the slit diaphragm on the capillary loops in the different groups. The same colors indicate the same Z-position within the total Z-volume of 4.5 µm. Data represent means ± SD *P < .05, **P < .001 by Student's *t*-test or by 1-way ANOVA followed by Bonferroni's *post hoc* test.

Treatment of rats with PAN nephrosis with either methylprednisolone or mifepristone (Fig. 3A) both significantly and similarly attenuated proteinuria (Fig. 3B). By light microscopy, PAN-treated rats showed normal glomerular morphology in all groups (Supplementary data, Fig. S2A). Podocyte stress, as assessed by desmin expression, was up-regulated in PAN-treated rats and significantly attenuated in both methylprednisolone- and mifepristone-treated rats (Fig. 2D and Supplementary data, Fig. S2B). Vehicle-treated mice had significantly lower average filtration slit density compared with mifepristone-treated mice (Fig. 3D and E).

#### Murine protein overload model

**Figure 4:**
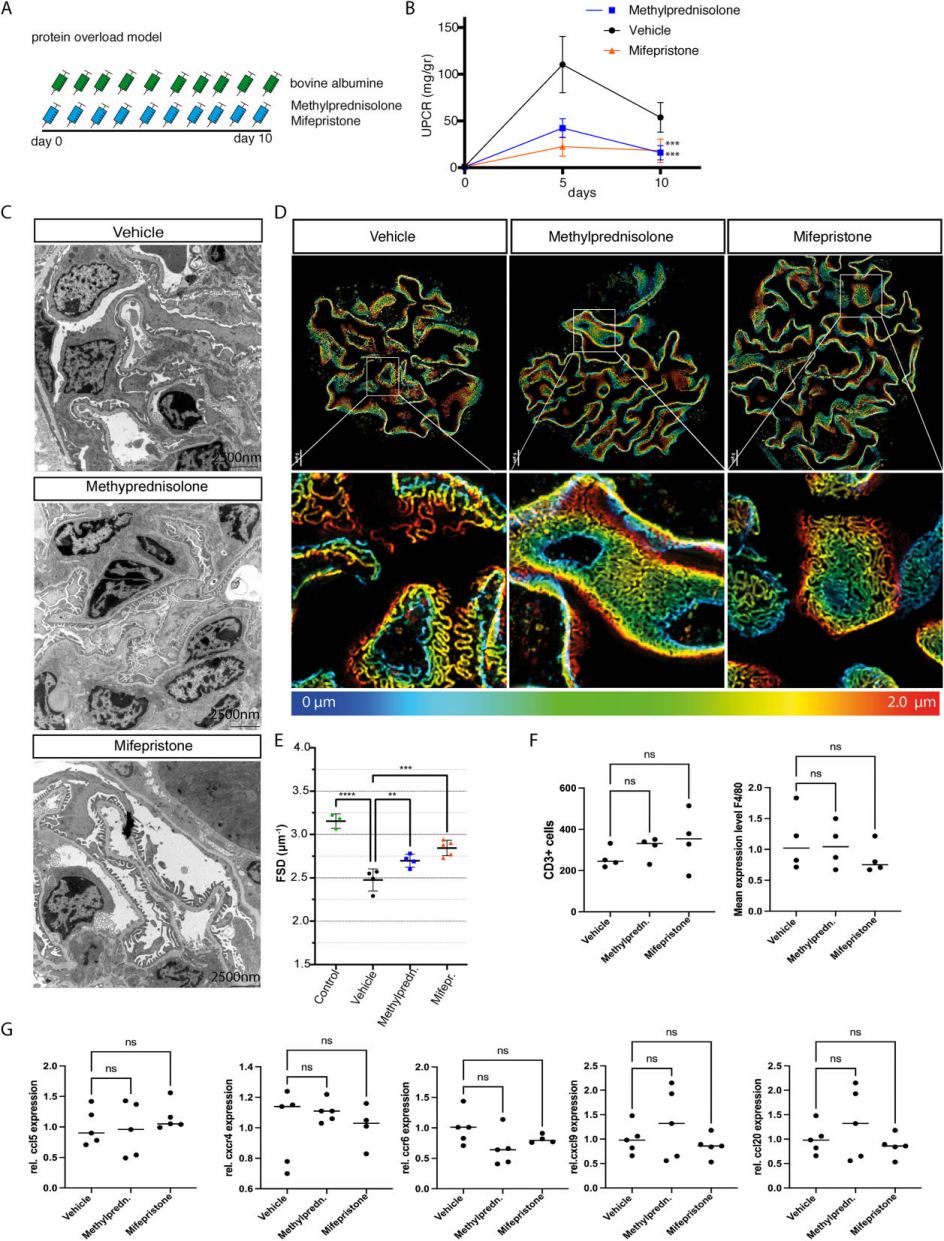
Pharmacological inhibition in murine protein-overload model. (**A**) Timeline of the experiment. Proteinuria was induced by bovine albumin injections [15 mg/g/body weight (bw) daily for 10 days (*n* = 6 per group)]. Mice were treated daily with either methylprednisolone 2.5 mg/kg bw or mifepristone 20 mg/kg bw or 0.9% NaCl (vehicle). (**B**) Proteinuria in vehicle-treated controls, and methylprednisolone- and mifepristone-treated mice. (**C**) Transmission electron microscopy of vehicle-treated controls, and methylprednisolone- and mifepristone-treated mice. Foot process effacement is present in vehicle-treated mice after induction of protein overload. Treatment with methylprednisolone or mifepristone preserved podocyte morphology and reduced podocyte effacement. (**D**) Images of 3D-reconstructed SIM volumes showing the spatial aspect of the meandering slit diaphragm on the capillary loops in vehicle-, mifepristione- and methylprednisolone-treated mice. In mifepristone- and methylprednisolone-treated mice, kidneys show a regular staining pattern with single foot process (FP) bridged by a meandering slit diaphragm in between. In the vehicle-treated mice the slit diaphragm appears less meandering and the foot process effaced. The same colors indicate the same Z-position within the total Z-volume of 4.5 µm. (**E**) Quantification of slit diaphragm density (FSD) in vehicle-, methylprednisolone- or mifepristone-treated mice with protein overload and healthy control mice. Both methylprednisolone and mifepristone resulted in increased slit diaphragm density compared with vehicle-treated mice. (**F**) Quantification of CD3+ cells in vehicle-, methylprednisolone- and mifepristone-treated mice (left graph). Quantification of F4/80 expression among the different experimental groups (right graph). (**G**) Relative mRNA expression of ccl5, cxcr4, ccr6, cxcl9 and ccl20 among the experimental groups. No significant differences were observed. Data are expressed as means ± SD. *ns*: not significant, **P < .01, ***P < .001, ****P < .0001 by one-way and two-way ANOVA followed by Bonferroni's *post hoc* test.

In the protein overload model (Fig. 4A), proteinuria peaked after 5 days (Fig. 3B). Kidney function and serum albumin remained unchanged (Supplementary data, Fig. S2C). By light microscopy, we observed mild mesangial expansion but no other glomerular alteration (Supplementary data, Fig. S2D). Treatment of these mice with high-dose methylprednisolone reduced proteinuria by about 60% (Fig. 4B). In parallel, we noted a reduction in podocyte effacement (Fig. 4C). Vehicle-treated mice had significantly lower average filtration slit density compared with methylprednisolone-treated mice (Fig. 4D and E).

To study the effects of partial GR inhibition, mice subjected to the protein-overload model were treated with mifepristone (Fig. 4B). This partial inhibitor does not induce a steroid deficiency syndrome in mice [11]. Mifepristone ameliorated proteinuria and foot process effacement to a similar extent to high-dose methylprednisolone (Fig. 4C–E). No difference was observed between male and female mice (Supplementary data, Fig. S2E).

To rule out systemic immunosuppressive effects of glucocorticoid receptor inactivation, we assessed the expression of inflammatory chemokines and performed immunostaining in the experimental groups (Fig. 4F and G). No differences were observed among the experimental groups. Additionally, spleen and kidney weights were compared among the experimental groups. Whereas spleen weight was significantly reduced in methylprednisolone-treated mice, it remained constant in mice with glucocorticoid receptor inactivation in comparison with vehicle-treated animals (Supplementary data, Fig. S2F). This confirmed previous reports that mifepristone does not exert systemic immunosuppressive or immunostimulatory effects [11, 21]. Kidney weight as a marker of renal edema was not affected by either methylprednisolone or mifepristone (Supplementary data, Fig. S2F).

#### Zebrafish model

**Figure 5:**
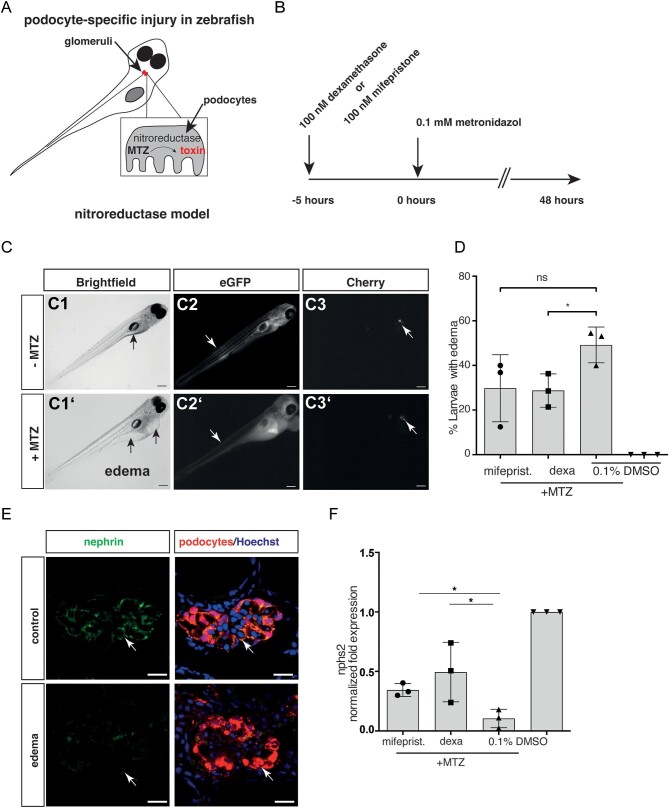
Pharmacological inhibition in zebrafish model. (**A**) Schematic of the zebrafish model. Zebrafish larvae (age 6 days post-fertilization) have a single glomerulus (in red) fused to a pair of tubules. If podocytes are partially depleted upon treatment with the prodrug MTZ, zebrafish larvae become proteinuric and display pericardial edema formation. (**B**) Timeline of the experiment. Animals were treated for 5 h with dexamethasone or mifepristone prior to addition of MTZ (i.e. induction of podocyte damage). (**C**) Edema formation in zebrafish larvae exposed to MTZ. (C1, C1′) Images of larvae with and without edema. Edematous larvae exhibit pericardial, yolk sac (arrows in C1 and C1′) and periorbital edema. (C2) Larvae with edema show decreased gc-eGFP fluorescence in the vasculature (arrows indicating segmental tail vessels in C2 and C2′), indicating a leaky filtration barrier, and decrease of mCherry fluorescence in the glomerulus (arrows in C3 and C3′). Scale bar: 100 µm. (**D**) Analysis of edema formation, a parameter of glomerular permeability defect, in mifepristone-, dexamethasone- and vehicle-treated zebrafish larvae. Edema formation is reduced by glucocorticoid agonism and antagonism. (**E**) Confocal laser scanning micrographs of glomeruli show decreased staining for nephrin (green) exclusively in larvae with edema compared with healthy control larvae (scale bar represents 10 µm). (**F**) Mean values of qRT-PCR for *nphs2* mRNA levels normalized to 18 s rRNA and compared with DMSO controls of whole larval lysates are shown. A 48-h exposure to 100 µM MTZ reduced *nphs2* mRNA levels to 0.06. Co-treatment with 100 nM dexamethasone or with 100 nM mifepristone resulted in an elevated *nphs2* expression (0.31 ± 0.05 and 0.25 ± 0.11, respectively), compared with control. The results of three independent experiments (*n* = 20 larvae per experiment) are expressed as mean mRNA levels ± SD. *P < .05, by one-way ANOVA followed by Bonferroni's *post hoc* test. Data represent means ± SD.

For further evaluation of the beneficial effects of mifepristone and glucocorticoids in podocyte injury, we used the transgenic NTR/MTZ model of podocyte injury in larval zebrafish. The Tg(*nphs2*: Eco.nfsB-mCherry) strain expresses the bacterial enzyme nitroreductase (NTR) and mCherry under podocyte-specific control of the nephrosis 2, idiopathic, steroid-resistant (podocin) *nphs2* promotor [14]. Application of MTZ to the medium of larvae induces podocyte apoptosis, proteinuria and pericardial and periocular edema [14] (Fig. 5A and B). For better visualization, we used a zebrafish line that expresses the transgene in the transparent *Casper* Background [Tg(*nphs2*: Eco.nfsB-mCherry), mitfa^w2/w2^; roy^a9/a9^], here referred to as *Cherry* [22].

Forty-eight hours of exposure of *Cherry* larvae to 100 µM MTZ, beginning at 4 days post-fertilization, resulted in pericardial and periocular edema (Fig. 5C). Compared with MTZ treatment alone, co-treatment with 100 nM mifepristone resulted in a significantly lower percentage of larvae with edema than in larvae co-treated with dexamethasone (25.8 ± 7.4% vs 28.2 ± 10.9%, *n* = 81). None of the 80 control-treated (0.1% DMSO) larvae developed edema (Fig. 5D).

Decreased staining for the slit diaphragm protein nephrin and impaired podocyte morphology, as demonstrated by endogenous nphs2: mCherry fluorescence, was found only in larvae exhibiting the edematous phenotype (Fig. 5E).

As a marker for the severity of podocyte injury, we measured *nphs2* mRNA levels by qRT-PCR. Upon 48 h MTZ treatment, *nphs2* mRNA levels were significantly decreased (0.06 ± 0.04) when compared with vehicle-treated (0.1% DMSO) control larvae (Fig. 5F). Compared with a single MTZ treatment, co-treatment with 100 nM mifepristone or dexamethasone significantly increased *nphs2* mRNA levels 4.17-fold and 5.16-fold, respectively (Fig. 5F).

### GR antagonism in nephrotic patients

Based on the preclinical data, three patients with primary FSGS or MCD were treated with 200 mg mifepristone three times daily on a compassionate-use basis (Table 2).

**Table 2:** Characteristics of patients.

	Patient 1		Patient 2				Patient 3		
	Before	After (8 weeks)	1. Relapse before/after (2 weeks)		2. Relapse before/after (2 weeks)		Before	After (10 weeks)	Normal range
Gender	Male		Male				Male		
Age, years	42		21				40		
Time since diagnosis of MCD/FSGS	5 days		19 years				27 years		
S-cholesterol (mg/dL)	226	170	250	242	202	223	345	251	<200
Triglycerides (mg/dL)	420	215	150	208	140	154	254	188	<200
S-total protein (g/dL)	6.1	7.4	7.4	7.2	7.2	6.9	6.3	6.8	6.6–8.7
AST (U/L)	26	19	30	39	18	19	21	17	<50
ALT (U/L)	34	26	54	19	26	22	21	18	<50
γ-GT (U/L)	30	29	21	19	17	16	52	16	<60
Comorbidities	Obesity and asthma						Diabetes mellitus type 2		
Co-medications	Ramipril, hydrochlorothiazide, fluticason/salmeterol						Ramipril		

S, serum; AST, aspartate transaminase; ALT, alanine transaminase; γ-GT, gamma-glutamyl transferase.

**Figure 6:**
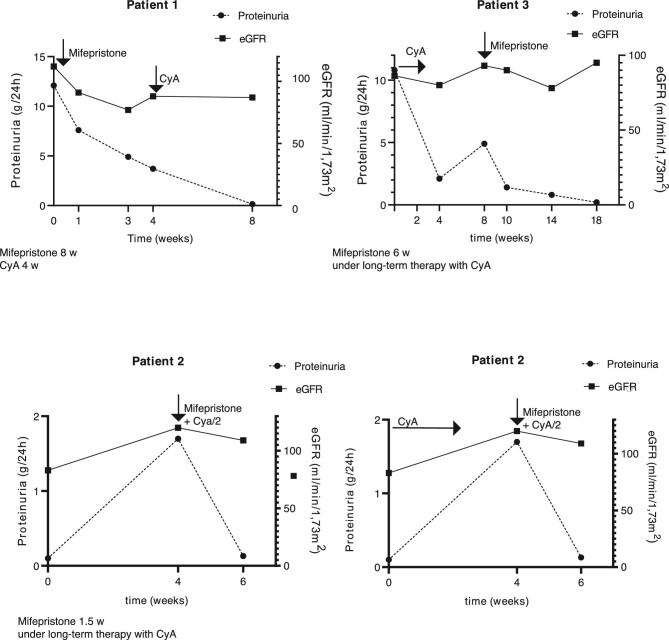
Glucocorticoid receptor antagonism in nephrotic patients. Course of proteinuria and eGFR in three patients treated with mifepristone. CyA/2: reduced-dose CyA.

Patient 1 was a 42-year-old nephrotic male, newly diagnosed with primary FSGS. Because of obesity (body mass index 38 kg/m^2^), first-line corticosteroid therapy was not considered. After 1 month of treatment with mifepristone, proteinuria had decreased from 12 to 4 g/day (67% reduction). At this time point, therapy with cyclosporine A (CyA) was also initiated to mediate an immunosuppressive effect against the permeability factor, which is thought to be derived from the immune system. Within one more month of dual treatment with mifepristone and cyclosporine, full remission (i.e. proteinuria <0.15 g/day) was achieved (Fig. 6).

Patient 2 was a 21-year-old-male with MCD diagnosed in childhood, suffering from frequent, steroid-sensitive relapses, and having been placed on maintenance therapy with CyA. Following another relapse despite ongoing CyA therapy, co-treatment with mifepristone was initiated for 10 days and proteinuria rapidly went into remission (92% reduction; proteinuria 0,1 g/day). However, treatment with mifepristone led to an about 2-fold increase in CyA blood trough levels. Upon a second relapse approximately 2 months later, the patient was again co-treated with mifepristone, but this time the CyA dose was adapted prospectively to maintain therapeutic trough levels of around 130 ng/mL. Again, proteinuria remitted to a similar extent as before within 10 days (Fig. 6).

Patient 3, a 40-year-old male with frequently relapsing MCD, diagnosed at the age of 13 years, developed a relapse of nephrotic syndrome despite maintenance therapy with CyA. As the patient refused corticosteroid therapy, mifepristone was given for about 6 weeks, and the CyA dose was prospectively adapted. Full remission of the nephrotic syndrome was achieved within 7 weeks (Fig. 6).

In all three patients, the medication was well tolerated and treatment-related adverse events have not been noted, in particular no abdominal or back pain, diarrhea, nausea or vomiting, headache or dizziness.

## DISCUSSION

Glucocorticoids represent the standard first-line treatment for patients with MCD/FSGS. However, some patients develop resistance to their action and, more importantly, glucocorticoids exhibit numerous side effects that are dose and time dependent. Hence, there is an unmet need for new therapeutic agents with fewer adverse events. In this study, we examined the role of the GR in proteinuric glomerular disease using genetic and pharmacologic manipulation. Genetic deletion of GR in epithelial cells, and use of GR antagonist mifepristone in different animal models was found to be as effective as the well-established role of GR activation in ameliorating proteinuria. Finally, selected patients with nephrotic proteinuria due to primary FSGS or MCD were treated on a compassionate-use basis with the GC receptor antagonist mifepristone which also satisfactorily translated our experimental findings to human condition.

The precise mechanism by which glucocorticoids elicit their renoprotective activity is only partially understood [23, 24]. Crucial components involved in the GR-mediated signaling pathway have been identified in cultured podocytes and previous studies have also provided evidence of functional GC signaling within podocytes [3, 25]. Regarding the mechanism through which both a GR agonist (methylprednisolone) and a GR antagonist (mifepristone) ameliorate proteinuria, it is plausible that mifepristone act as a partial agonist, thereby activating GR to some extent. This suggestion is supported by evidence indicating that mifepristone moderately enhances GR phosphorylation and activation, which aligns with its known partial agonistic properties [25, 26].

Our findings in mice with a genetic inactivation of the GR in renal epithelial cells are in contrast to those of GR inactivation in podocytes by Zhou *et al*. [27]. They used a podocin-Cre transgene and described that loss of the podocyte GR aggravates rather than ameliorates proteinuria following injury induced by lipopolysaccharide (LPS) or the nephrotoxic serum nephritis model. Presently, these discrepant results are difficult to reconcile, but of course may relate to the usage of different knock-out mouse lines and different models of injury.

Importantly, our highly consistent findings based on pharmacological and genetic inhibition of the GR in attenuating proteinuria in three animal models strongly support that specific inactivation or antagonism of the GR in proteinuric diseases can be used to improve nephrotic syndrome caused by non-inflammatory glomerular disease.

Importantly, we also validated the above concept in three patients with nephrotic-range proteinuria. In all of them, mifepristone was combined with a calcineurin inhibitor. Given that calcineurin inhibitors such as CyA have direct effects on podocytes [28], additive or synergistic effects of both agents on podocytes are conceivable. It could also be argued that the proteinuric effects relate to mifepristone-associated increases of CyA in serum. However, we have adapted the doses of CyA with mifepristone to achieve stable therapeutic CyA levels; the anti-proteinuric effect of mifepristone was maintained. Finally, spontaneous remissions in these three reported patients cannot be definitively excluded; however, since three out of three patients responded similarly within a relatively short period of time, this possibility seems less likely.

Given that treatment with mifepristone is associated with fewer adverse effects compared with high doses of glucocorticoids [29], it may be considered an alternative therapeutic option for patients with proteinuric glomerular disease, particularly for those patients who are resistant to glucocorticoids, steroid-dependent or who cannot receive glucocorticoids due to other comorbidities or previous steroid-related complications. Common side effects of mifepristone include abdominal pain, fatigue and vaginal bleeding [30]. Serious adverse events, such as heavy bleeding, are very rare and have only been reported after medical abortion.

Mifepristone is a partial GR antagonist but also a steroidal antiprogestogen and antiandrogen to a much lesser extent [15]. Thus, potential off-target effects of the pharmacological intervention with mifepristone, which might have contributed to the effects we observed, cannot be excluded. However, our findings using the genetic deletion of GR confirm that this possibility seems less likely and suggest that inactivation of GR is a beneficial route for reducing proteinuria. Of note, no differences were observed between male and female animals. Furthermore, it is quite possible that administration of mifepristone results in hormonal imbalance by altering the hypothalamic–pituitary–adrenal axis, resulting in the change in levels of cortisol, corticotrophin-releasing hormone and adrenocorticotrophic hormone [31]. Reduced conversion of cortisol into cortisone in kidneys could lead to increased activation on the mineralocorticoid receptor (MR). However, as reviewed by us [32] and demonstrated by others, overactivation of the MR can have injurious effects on podocytes, which would in contrast with our findings result in opposing effects to the proteinuria reducing effects of mifepristone. Moreover, kidneys have typically high expression of 11β-hydroxysteroid dehydrogenase type 2, which prevents the overactivation of the MR [33]. Inactivation or reduced activity of this enzyme can occur in the kidneys in certain disease conditions, but typically cortisol can be converted efficiently to cortisone, which does not bind to MR.

As our genetic approach to inactivate the GR, i.e. the Pax8-Cre/GR^fl/fl^ transgenic mice, is not podocyte specific, we acknowledge that benefits in proteinuric models induced in these mice may not only result from altered podocyte responses but also altered responses of PECs or proximal tubular cells. Particularly, we cannot exclude that the reabsorption of protein in tubules is altered in GR-deficient tubular cells leading to changes in proteinuria. However, this would more likely result in aggravation rather than amelioration of proteinuria. In addition, immunostaining and transmission electron microscopy did not show any significant tubular or PEC activation/injury in either control or transgenic mice. With respect to PECs, it is known that PECs become activated in the protein-overload model [34], but antiproteinuric effects of reduced PEC activation would be difficult to explain on the basis of our current understanding of the pathophysiology of proteinuria. Moreover, the possibility of hormonal imbalance resulting from adrenal GR insufficiency due to the GR allele excision is not likely in the Pax8-Cre/GR^fl/fl^ transgenic mice as Pax8 inactivation (as well as Pax2) has been shown to result in normal adrenal gland formation [35]. This study demonstrates that while the *Pax2*^+/−^*Pax8*^−/−^ embryos fail to develop a kidney, ureter and genital tract (vas deferens), the adrenal gland, testis and bladder form normally. Thus, it is unlikely that the GR alleles are excised in the adrenal gland in our model. Furthermore, it is important to acknowledge the limitations of using the protein overload as a model in our study. While we recognize that protein overload may not precisely mimic the pathophysiology of glomerular diseases in humans, we selected this model due to the unavailability of a suitable mouse model. Specifically, we could not use the adriamycin model due to its potential insensitivity to steroid treatment.

In summary, our results show that the GR plays a direct role in renal epithelial cells, including podocytes in particular, and this benefit is maintained across species. This suggests that GR inhibition may be a potential new therapeutic approach in patients with MCD and primary FSGS, and perhaps other proteinuric diseases. Our study, in particular our pilot observations in three patients lay the basis for a more systematic analysis of GR antagonism in patients with non-inflammatory proteinuric renal diseases. This novel therapeutic approach may be particularly important in patients with contraindications to high doses of glucocorticoids such as those with comorbid diabetes, infections, obesity and psychiatric conditions.

## Supplementary Material

gfad254_Supplemental_Files

## Data Availability

All data are available in the main text or Supplementary data. The data will be shared on reasonable request to the corresponding author.
