# Supplementary material for: Inhibition of the glucocorticoid receptor attenuates proteinuric kidney diseases in multiple species
Source: Nephrol Dial Transplant. 2023 Nov 30;39(7):1181–93. doi: 10.1093/ndt/gfad254 (PMC11210988; doi:10.1093/ndt/gfad254)
Supplement: gfad254_Supplemental_Files [file gfad254_supplemental_files.zip › Suppl. fig1.docx]

**Suppl. Figures**

**Suppl. Fig. 1. (A)** Proteinuria in male and femali transgenic and control mice. No significant difference was found between male and female in both experimental groups. **(B)** Renal functional parameters, serum albumin and cholesterol levels in GR-KO mice and controls at Day 10 after disease induction. **(C)** Representative PAS-stained paraffin sections from control and GR-KO mice. Data are expressed as means ± SD; *ns: not significant,* **p<0.01, by Student’s t-test or by 1-way ANOVA followed by Bonferroni’ post-hoc test.

**Suppl. Fig. 2. Pharmacological inhibition of GR**. (**A**) Representative PAS-stained paraffin sections from PAN-treated rats showed normal glomerular morphology (**B**) Representative desmin immunohistochemistry. (**C**) Renal functional parameters (serum creatinine and urea), serum albumin and cholesterol levels at Day 10 in the protein-overload model. (**D**) Representative PAS-stained paraffin sections from control and methylprednisolone- and mifepristone-treated mice. (E) Proteinuria in male and female mifepristone-treated mice. No significan t differences were found between male and female mice. (E) Spleen-to-body-weight ratios as a surrogate parameter for systemic inflammation were reduced significantly by high-dose methylprednisolone treatment but not by mifepristone. Kidney-to-body-weight ratios remained unchanged among the experimental groups. Data are expressed as means ± SD; *ns: not significant,* **p<0.01, by Student’s t-test or by 1-way ANOVA followed by Bonferroni’ post-hoc test.
