# Supplementary figures and images for: Inhibition of the glucocorticoid receptor attenuates proteinuric kidney diseases in multiple species
Source: Nephrol Dial Transplant. 2023 Nov 30;39(7):1181–93. doi: 10.1093/ndt/gfad254 (PMC11210988; doi:10.1093/ndt/gfad254)

suppl. Figure 1

A

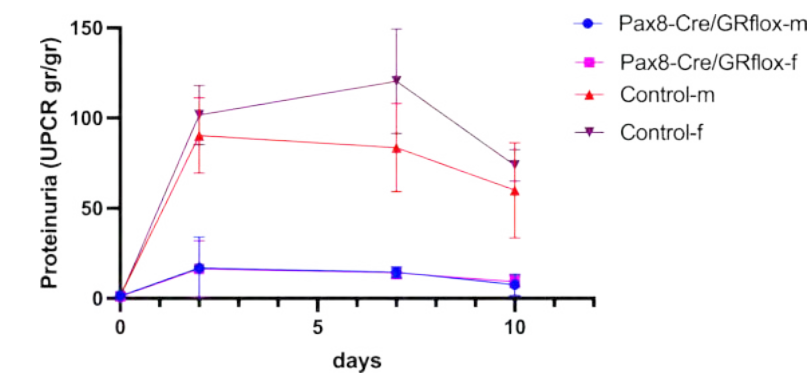

B

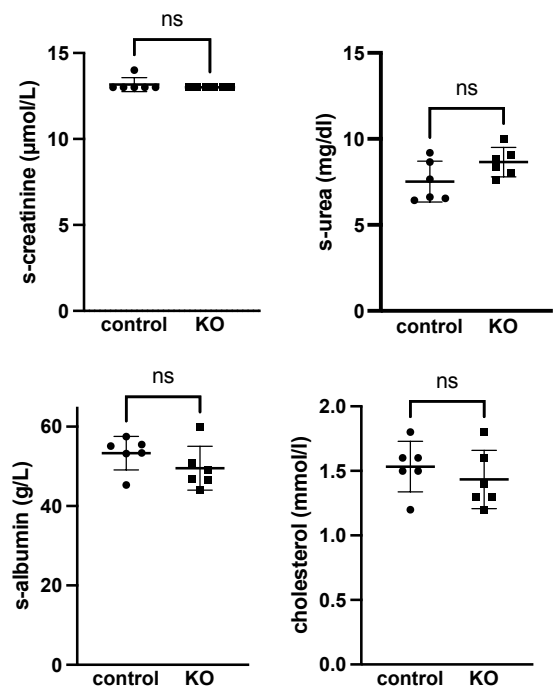

C

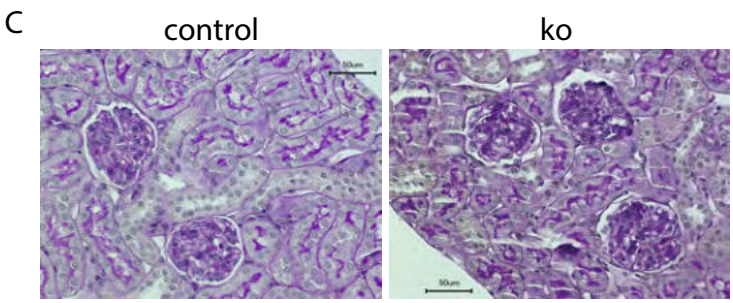

F

# suppl. Figure 2

A

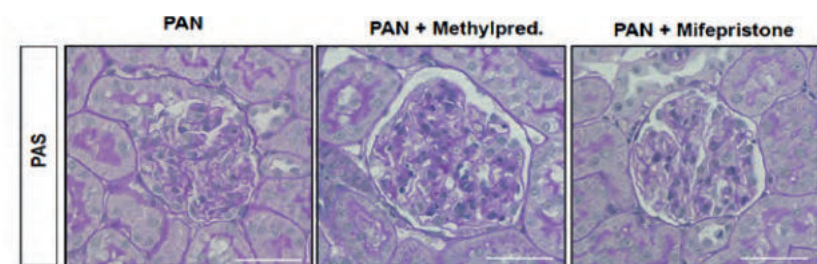

B

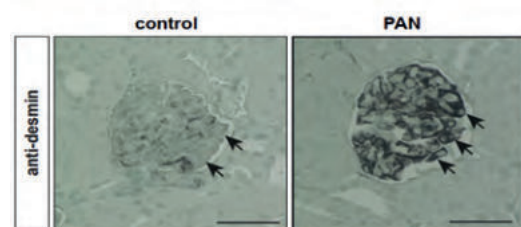

C

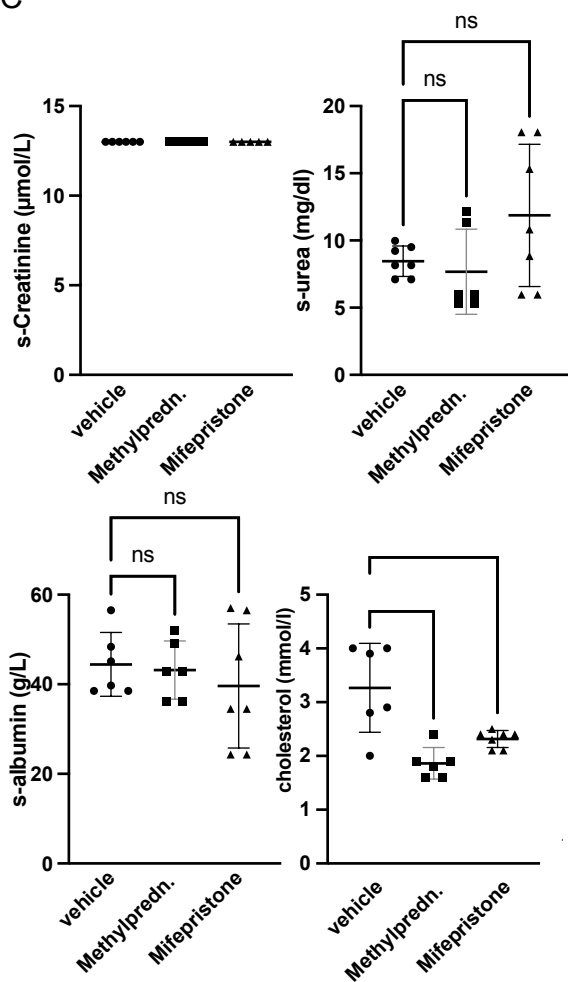

D

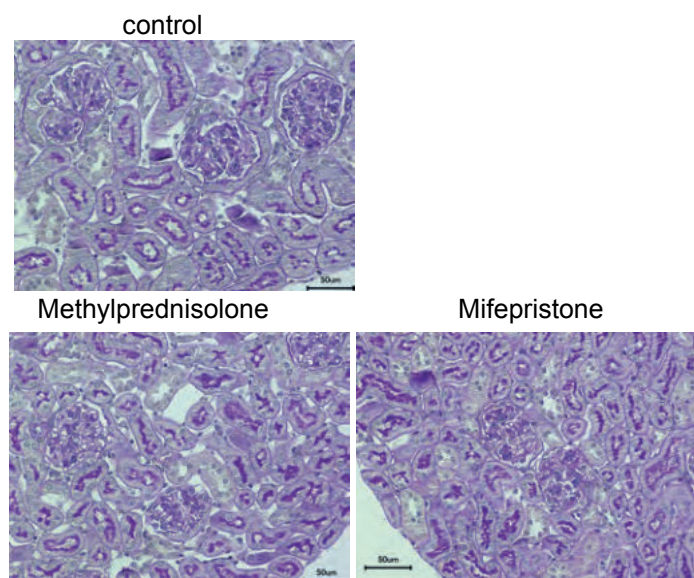

F

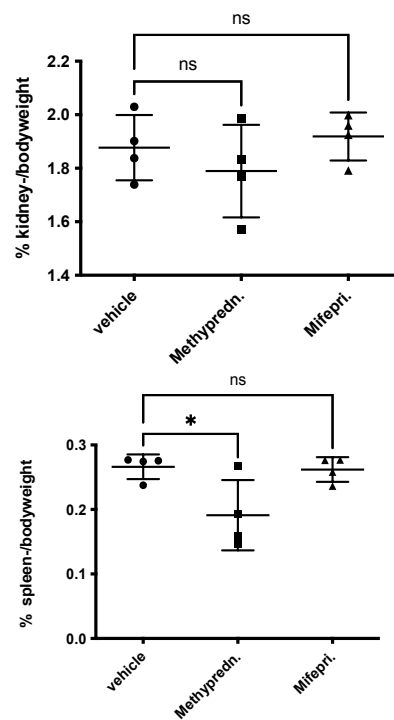

E

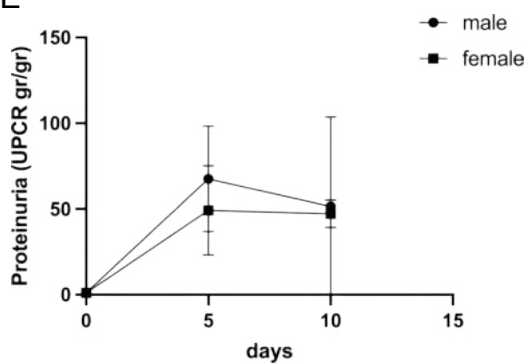

Supplement: gfad254_Supplemental_Files [file gfad254_supplemental_files.zip › submission_2.pdf]
